# circ_0038467 promotes PM2.5-induced bronchial epithelial cell dysfunction

**DOI:** 10.1515/med-2021-0213

**Published:** 2021-06-10

**Authors:** Xuan Jin, Li Wang, Mingzhu Yang

**Affiliations:** Department of Pediatrics, Affiliated Hospital of Shaanxi University of Traditional Chinese Medicine, No. 2, Weiyang West Road, Xianyang 712000, Shaan’xi, China; Department of Pediatrics, Xianyang Central Hospital, Xianyang, Shaan’xi, China; Department of Clinical laboratory, Affiliated Hospital of Shaanxi University of Traditional Chinese Medicine, Xianyang, Shaan’xi, China

**Keywords:** circ_0038467, miR-138-1-3p, NF-κB pathway, BEAS-2B, lung dysfunction

## Abstract

**Purpose:**

This study was to explore the toxicological mechanisms by which PM2.5 causes lung dysfunction.

**Methods:**

The expression of circ_0038467 and miR-138-1-3p in PM2.5-induced human bronchial epithelial cell line BEAS-2B was detected by RT-qPCR. The effects of circ_0038467 and miR-138-1-3p on proliferation, apoptosis, and inflammatory cytokines (IL-6 and IL-8) in PM2.5-induced BEAS-2B were determined using cell counting kit-8, flow cytometry, western blot, and enzyme-linked immunosorbent assay, respectively. The levels of nuclear factor kappa B (NF-κB) pathway-related protein were also analyzed by western blot. The binding interaction between circ_0038467 and miR-138-1-3p was confirmed by dual-luciferase reporter assay and RNA immunoprecipitation assay and pull-down assay.

**Results:**

circ_0038467 expression was increased by PM2.5 treatment in BEAS-2B cells in time- and dose-dependent methods, and knockdown of circ_0038467 reversed PM2.5-triggered BEAS-2B cell death and inflammatory response. miR-138-1-3p was decreased by PM2.5 treatment, and restoration of miR-138-1-3p attenuated PM2.5-induced BEAS-2B cell injury. In a mechanical study, we found circ_0038467 directly bound to miR-138-1-3p, and further rescue experiments exhibited miR-138-1-3p inhibition partially overturned the regulatory functions of circ_0038467 knockdown in PM2.5-induced BEAS-2B cells.

**Conclusion:**

circ_0038467 provided a potential therapeutic strategy for future clinic intervention in air pollution-triggered lung dysfunction.

## 1 Introduction

Increasing evidence has shown that long-term air pollution can damage bronchial mucosa and induce bacterial invasion and inflammation reaction of airway and pulmonary, which lead to severe lung disorders in person; among them, the impact is particularly serious in children due to the incomplete development of the respiratory tract [1,2]. With the development of industry, air pollutant particle matters (PM) with an aerodynamic diameter less than 2.5 µm (PM2.5) have been revealed to be associated with the elevation of respiratory morbidity and mortality [3,4]. Compared with Western countries, the study into the hazard of PM2.5 in China has just started. PM2.5 is reported to mainly occur in the Beijing–Tianjin–Hebei Economic Zone, the Yangtze River Delta, the Pearl River Delta region, the three northeastern provinces, the Sichuan Basin, and other densely populated areas, and seriously affects public health both physically and emotionally [5]. PM2.5 is a mixture of solid and liquid particles, including black carbon, sulfate, nitrate, metals, polycyclic aromatic hydrocarbons, and automobile exhaust particles, with the characteristics of small particle size, large surface area, and toxin absorption ability [6]. It can be inhaled and deposited in the epithelium cells of the alveolar, thus aggravating airway inflammation, oxidative injury, aging, and immoderately death of cells [7,8,9]. Therefore, further investigations on the mechanisms underlying the toxicity of PM2.5 are necessary for developing effective therapies of lung dysfunction.

Circular RNAs (circRNAs) are defined as a novel type of transcripts with ring structures from the ligation of exons, introns, or both that generally make them resist to RNase R decay [10,11]. circRNAs are abundantly expressed in the eukaryotic genome, exhibit species-, tissue-, cell-, and disease-specific expression patterns, and have been demonstrated to play key roles in cellular crucial biological processes [12,13,14]. Importantly, a growing number of studies have indicated that circRNAs are intensively associated with different respiratory diseases, such as lung cancer, pulmonary tuberculosis chronic obstructive pulmonary disease (COPD), and pulmonary hypertension [15,16]. circ_0038467 was a novel identified circRNA, produced from the back-splicing of ubiquinol-cytochrome-c reductase core protein 2 (UQCRC2) gene, and was found to be upregulated in lipopolysaccharide (LPS)-induced human bronchial epithelial (HBE) cells and reinforced LPS-induced cell inflammatory injury [17]. Thus, we suspected that circ_0038467 might also involve in PM2.5-induced HBE cell dysfunction.

Here, this work aimed to investigate the expression pattern and pathological role of circ_0038467 in PM2.5-induced HBE cell line BEAS-2B and explored the potential regulatory network underlying circ_0038467 in PM2.5-evoked BEAS-2B injury.

## 2 Materials and methods

### 2.1 PM preparation

PM2.5 stock solution was prepared by dissolving standard reference PMs (SRM 1648a) (50 mg; NIST, Gaithersburg, MD, USA) in 500 µL phosphate buffer solution (PBS) and 500 µL dimethyl sulfoxide (DMSO) and subjected to ultrasound oscillation on ice for 30 min through ultrasonography. Then Toxinsensor™ Endotoxin Detection System (GenScript Biotech, Nanjing, China) was applied to test endotoxin levels.

### 2.2 Cell culture and transfection

BEAS-2B cells were obtained from Beijing Institute for Cancer Research Collection (Beijing, China) and cultured in bronchial epithelial cell basal medium (BEBM) (Lonza, Switzerland) with 5% CO_2_ at 37°C. BEAS-2B cells were treated with PM2.5 at particulate doses (0, 50, 75, and 100 μg/mL) for different time-periods (0, 24, 48, and 72 h) to establish a PM2.5-induced BEAS-2B cell injury model.

The small interfering RNA (siRNA) targeting circ_0038467 (si-circ_0038467) with negative control (si-NC), the mimic, or inhibitor of miR-138-1-3p (miR-138-1-3p or anti-miR-138-1-3p) with negative control (miR-NC or anti-miR-NC) were obtained from GenePharma (Shanghai, China). Transfection of BEAS-2B cells with si-circ_0038467 or si-NC (40 nM), and miR-138-1-3p or miR-NC (10 nM), and anti-miR-138-1-3p or anti-miR-NC (10 nM) was performed with Lipofectamine 2000 transfection reagent (Invitrogen, Carlsbad, CA, USA).

### 2.3 RNA extraction and RNase R digestion

TRIzol reagent (Invitrogen) was used to isolate total RNA from BEAS-2B cells. Then 100 µg of RNA extracts were treated without or with RNase R (Qiagen, Tokyo, Japan) for 15 min at 37°C; thereafter, the resulting RNAs were purified by using RNeasy MinElute Cleanup Kit (Qiagen).

### 2.4 Reverse transcriptase PCR (RT-qPCR)

Approximately 1 μg of RNA extracts was used to reversely transcribe to complementary DNAs (cDNAs) using a script RT reagent kit (Qiagen). Subsequently, synthesized cDNAs were qualified using SYBR-Green PCR kit (Qiagen) on an ABI 7500 Real-Time PCR system. The U6 or glyceraldehyde 3-phosphate dehydrogenase (GADPH) was employed as an internal control and the 2^−ΔΔCt^ method was used to calculate the relative fold changes. The following primers were used: circ_0038467: F, 5′-CTGAACGTTCTCTCAGCCCAG-3′ and R, 5′-GGTCAGCTACTTCCCAACGA -3′; GADPH: F 5′-GGTGAAGGTCGGAGTCAAC-3′ and R 5′-AGAGTTAAAAGCAGCCCTGGTG-3′; U6: F, 5′-CTCGCTTCGGCAGCACA-3′ and R, 5′-AACGCTTCACGAATTTGCGT-3′; UQCRC2: F, 5′-AGCAACACCACCAGCCATC-3′, R, 5′-TAAATCCCAAAGAGTCCAG-3′; miR-138-1-3p: F, 5′-GCCGAGGCTACTTCACAACACC-3′ and R, 5′-CAGTGCAGGGTCCGAGGTAT-3′.

### 2.5 Cell counting kit-8 (CCK-8) assay

BEAS-2B cells (5,000/well) seeded in 96-well plates were transfected with assigned vectors for 48 h, followed by exposure with 75 μg/mL PM2.5. Forty-eight hours later, per well was interacted with 10 μL CCK-8 solution (5 mg/mL, Sigma-Aldrich, Irvine, Ayrshire, UK) for 4 h. The absorbance at 450 nm of each well was tested using a microplate reader.

### 2.6 Lactate dehydrogenase release (LDH) assay

Following transfection, BEAS-2B cells (1 × 10^4^) were exposed to 75 μg/mL PM2.5 for 48 h. Then the culture supernatants of BEAS-2B cells were collected and LDH release was detected using an LDH assay kit (Sigma-Aldrich) referring to the protocol’s instructions.

### 2.7 Flow cytometer

Following transfection with assigned vectors, BEAS-2B cells were exposed to 75 μg/mL PM2.5 for 48 h. Afterwards, cells (1 × 10^6^ cells/mL) were resuspended in 1X Annexin V binding buffer, and then double-stained with FITC-Annexin V (10 μL) and propidium iodide (10 μL) for 15 min, and analyzed using a FACScan flow cytometer (BD Biosciences, San Jose, CA, USA).

### 2.8 Western blot

Following transfection and/or treatment, proteins were isolated from transfected BEAS-2B cells through using RIPA lysis buffer (Beyotime, Shanghai, China). After the qualification of protein concentration with the bicinchoninic acid assay, approximately 30 μg of extracted protein was loaded and subjected to 10% sodium dodecyl sulfate polyacrylamide gel electrophoresis and transferred onto polyvinylidene difluoride (PVDF) membranes. After that, the membranes were incubated with primary antibodies recognizing B-cell lymphoma-2 (Bcl-2) (1:3,000, ab692), Bcl-2-associated X (Bax) (1:3,000, ab32503), phosphorylated (p)-p65 (1:5,000, ab86299), p-IκB-α (1:5,000, ab133462), interleukin-6 (IL-6) (1:2,000, ab6672), IL-8 (1:2,000, ab18672), and GAPDH (1:10,000, ab181602) overnight at 4°C, followed by interaction with HRP-conjugated secondary antibodies (1:1,000, ab205719) for 2 h at 37°C. All antibodies were obtained from Abcam (Cambridge, MA, USA). Protein bands were visualized by electrochemiluminescence (ECL).

### 2.9 Enzyme-linked immunosorbent assay (ELISA)

The concentrations of IL-6 and IL-8 from the culture supernatants of BEAS-2B cells after appropriate transfection and/or treatment were analyzed using commercial IL-6 and IL-8 ELISA kits (R&D Systems, Minneapolis, Minnesota, USA) referring to the instructions of protocol.

### 2.10 Subcellular fractionation

Cytoplasmic and Nuclear RNA Purification Kit (Thermo Fisher Scientific, Inc., Waltham, MA, USA) was employed to isolate and purify the RNA from nuclear and cytoplasm fractions. Next, the relative expression levels of circ_0038467, GAPDH, and U6 were determined by RT-qPCR assay.

### 2.11 RNA immunoprecipitation (RIP) assay

BEAS-2B cells were lysed by using RIP buffer, and then cell lysate was incubated with RIPA buffer containing magnetic beads conjugated with human anti-Ago2 antibody or normal mouse anti-IgG (Millipore, Billerica, MA, USA), followed by interaction with Proteinase K (Millipore). Finally, the immunoprecipitated RNA was extracted and circ_0038467 levels were analyzed using RT-qPCR assay.

### 2.12 Dual-luciferase reporter assay

The sequences circ_0038467 that contained the miR-138-1-3p binding sites were cloned into the pmirGLO luciferase vector (Promega, Madison, WI, USA) to construct pmirGLO-wild-type/mutant-circ_0038467, named circ_0038467-WT/circ_0038467-MUT. Subsequently, miR-138-1-3p mimic or miR-NC were transfected into BEAS-2B cells that were transfected with circ_0038467-WT/MUT. Luciferase activity was determined using a dual-Luciferase reporter assay kit (Promega) and normalized by Renilla luciferase activity.

### 2.13 Pull-down assay

Biotin-labeled miR-138-1-3p (Bio-miR-138-1-3p), Bio-miR-138-1-3p-MUT, and Bio-miR-NC generated by GenePharma were transfected into BEAS-2B cells. Forty-eight hours later, cells were lysed and incubated with Dynabead M-280 streptavidin beads (Invitrogen) overnight. The RNA complexes bound to the beads were eluted, extracted, and subjected for RT-qPCR analysis.

### 2.14 Statistical analysis

Data from thrice-repeated experiments were presented as mean ± standard deviation (SD). Comparisons of parameters were analyzed using Student’s *t*-test, Mann–Whitney *U* tests, and one-way analysis of variance (ANOVA) as appropriate. The *P* < 0.05 was indicated as statistically significant.

## 3 Results

### 3.1 PM2.5 accelerated circ_0038467 expression in BEAS-2B cells

**Figure 1:**
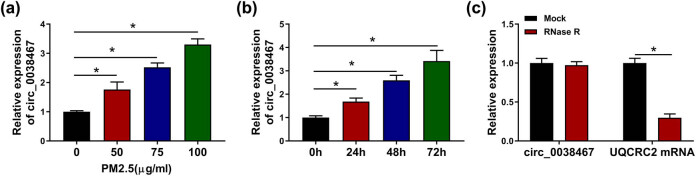
PM2.5 accelerated circ_0038467 expression in BEAS-2B cells. (a and b) RT-qPCR analysis of circ_0038467 expression in BEAS-2B cells exposed to a range of concentrations of PM2.5 (0, 50, 75, and 100 µg/mL) over different time-periods (0, 24, 48 and 72 h). (c) RT-qPCR analysis of the expression of circ_0038467 and linear transcript UQCRC2 after RNase R treatment. *N* = 3, *P < 0.05.

To investigate the potential effects of circ_0038467 on PM2.5-induced injury of HBE cells, first of all, the different expression of circ_0038467 in HBE cells was detected. BEAS-2B cells were exposed to a range of concentrations of PM2.5 (0, 50, 75, and 100 µg/mL) for 48 h; the expression of circ_0038467 in HBE cells was found to be increased in a dose-dependent manner (Figure 1a). Moreover, circ_0038467 expression was also time-dependently upregulated in HBE cells by 75 µg/mL PM2.5 exposure at 0, 24, 48, and 72 h (Figure 1b). All these data suggested that aberrant expression of circ_0038467 might be associated with PM2.5-induced BEAS-2B cell dysfunction. Afterwards, RNase R assay was performed, and we found circ_0038467 was resistant to RNase R digestion, while the linear transcript was markedly digested by RNase R treatment (Figure 1c), indicating that circ_0038467 was indeed circular transcript.

### 3.2 circ_0038467 knockdown reduced PM2.5-induced cell death and inflammatory response in BEAS-2B cells

**Figure 2:**
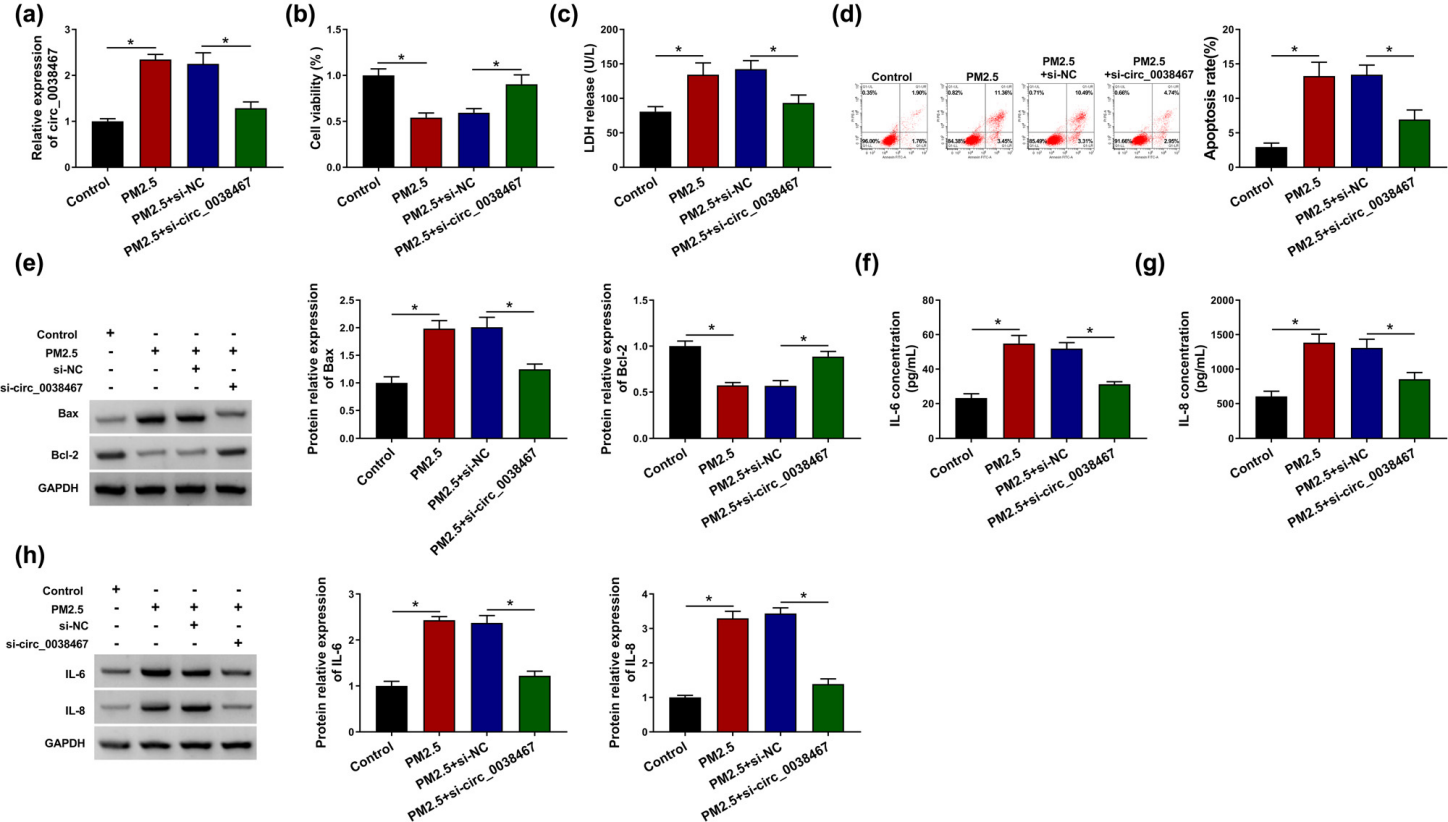
circ_0038467 knockdown reduced PM2.5-induced cell death and inflammatory response in BEAS-2B cells. BEAS-2B cells were transfected with si-circ_0038467 or si-NC, then treated with 75 μg/mL PM2.5 for 48 h. (a) RT-qPCR analysis of circ_0038467 expression in BEAS-2B cells. (b) CCK-8 assay of BEAS-2B cell viability. (c) LDH assay of the release of LDH in BEAS-2B cells. (d) Flow cytometry of BEAS-2B cell apoptosis. (e) Western blot analysis of Bax and Bcl-2 expression in BEAS-2B cells. (f–h) Levels detection of IL-6 and IL-8 expression in BEAS-2B cells using ELISA and western blot analyses. *N* = 3, *P < 0.05.

To get insights into the effect of circ_0038467 on PM2.5-induced HBE cell injury, BEAS-2B cells were transfected with si-circ_0038467 or si-NC, then treated with 75 μg/mL PM2.5 for 48 h. The introduction of si-circ_0038467 obviously decreased PM2.5-induced upregulation of circ_0038467 expression (Figure 2a). From the CCK-8 assay, we found that PM2.5 exposure remarkably suppressed cell viability of BEAS-2B cells, while this condition was reversed by circ_0038467 knockdown (Figure 2b). LDH assay showed knockdown of circ_0038467 decreased PM2.5-induced LDH release in BEAS-2B cells (Figure 2c), revealing that downregulation of circ_0038467 suppressed PM2.5-induced damage to the membrane of BEAS-2B cells. Meanwhile, we verified that PM2.5 exposure increased the apoptosis rate of BEAS-2B cells by elevating Bax expression and decreasing Bcl-2 expression, while downregulation of circ_0038467 abated PM2.5-induced cell apoptosis (Figure 2d and e). Additionally, the results from ELISA and western blot analyses exhibited that PM2.5 exposure significantly increased IL-6 and IL-8 expression in BEAS-2B cells, which were attenuated by circ_0038467 silencing (Figure 2f–h). Taken together, downregulation of circ_0038467 blocked PM2.5-induced cell death and inflammatory response of BEAS-2B cells.

### 3.3 miR-138-1-3p was a target of circ_0038467

**Figure 3:**
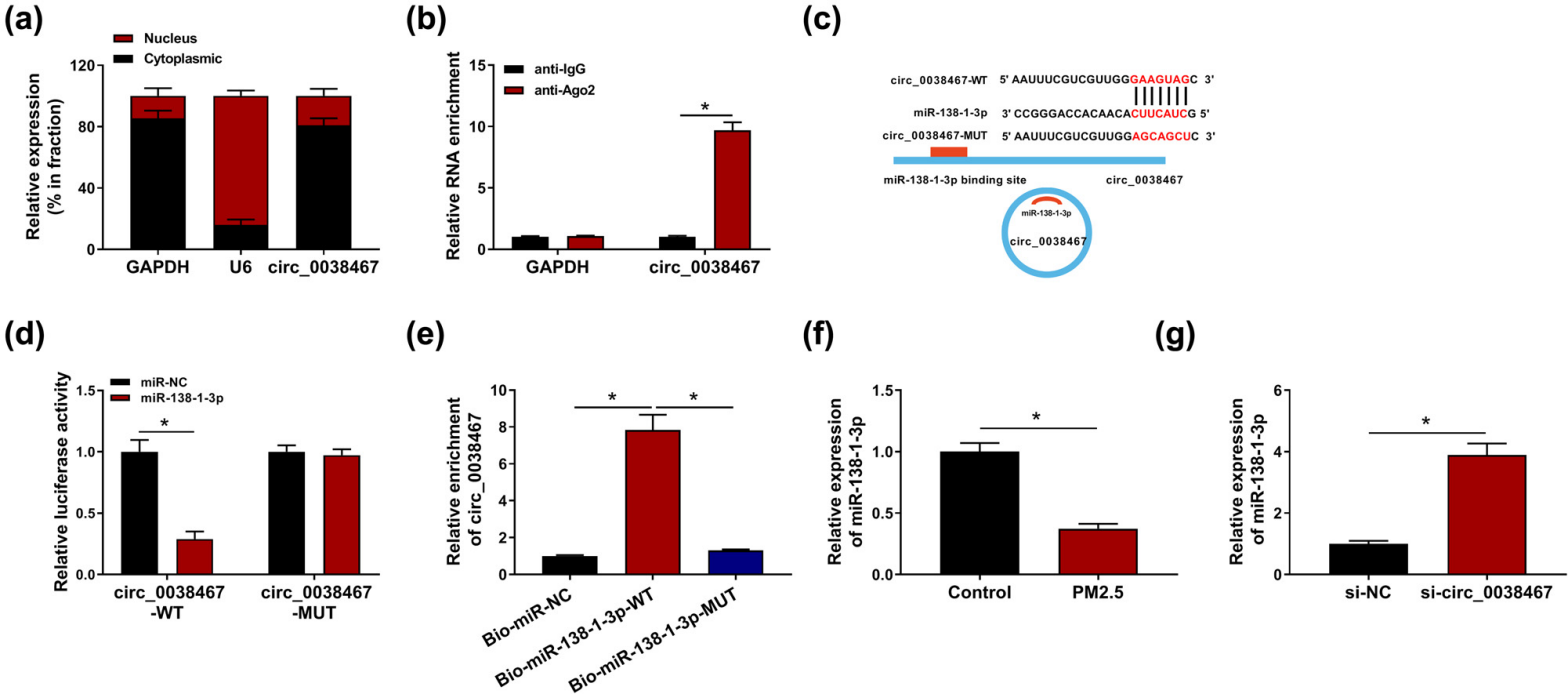
miR-138-1-3p was target of circ_0038467. (a) circ_0038467 level by RT-qPCR in the nuclear and cytoplasm fractions of BEAS-2B cells. (b) circ_0038467 level by RT-qPCR in BEAS-2B cells after RIP assay. (c) The potential binding sites of circ_0038467 and miR-138-1-3p. (d) Luciferase activities detection in BEAS-2B cells co-transfected with the reporter plasmid and the indicated miRNAs using the dual-luciferase reporter assay. (e) The enrichment of circ_0038467 by RT-qPCR in BEAS-2B cells pulled down by Bio-miR-138-1-3p. (f) RT-qPCR analysis of miR-138-1-3p expression in BEAS-2B cells treated with or without PM2.5. (g) RT-qPCR analysis of miR-138-1-3p expression in BEAS-2B cells transfected with si-NC or si-circ_0038467. *N* = 3, *P < 0.05.

The subcellular localization of circ_0038467 was investigated, and results showed circ_0038467 was highly enriched in the cytoplasm fraction in BEAS-2B cells (Figure 3a). Besides, RIP assay suggested circ_0038467 could be enriched by anti-Ago2 antibody relative to IgG antibody (Figure 3b). Thus, we suspected that circ_0038467-mediated regulatory functions might operate through a sponge mechanism. Through the using of online databases CircInteractome, the potential underlying microRNAs (miRNAs) that could be interacted with circ_0038467 was searched, and miR-138-1-3p was identified to have putative binding sites of circ_0038467 (Figure 3c). Then dual-luciferase reporter assay was implemented in BEAS-2B cells, and we observed that the luciferase activity of wild-type reporter was significantly reduced by miR-138-1-3p overexpression, while there was no change in mutated reporter when miR-138-1-3p was upregulated (Figure 3d). Moreover, pull-down assay exhibited that circ_0038467 levels in BEAS-2B cells pulled down using Bio-miR-138-1-3p were higher than those in cells pulled down by Bio-miR-NC or Bio-miR-138-1-3p-MUT (Figure 3e). All these results confirmed that circ_0038467 directly bound to miR-138-1-3p in BEAS-2B cells. Subsequently, the different expression of miR-138-1-3p in BEAS-2B cells was explored; RT-qPCR analysis suggested miR-138-1-3p expression was decreased by PM2.5 exposure (Figure 3f), but was increased by circ_0038467 knockdown (Figure 3g). Overall, we confirmed that miR-138-1-3p was a target of circ_0038467 and might be related to PM2.5-induced BEAS-2B cell dysfunction.

### 3.4 miR-138-1-3p alleviated PM2.5-induced cell death and inflammatory response in BEAS-2B cells

**Figure 4:**
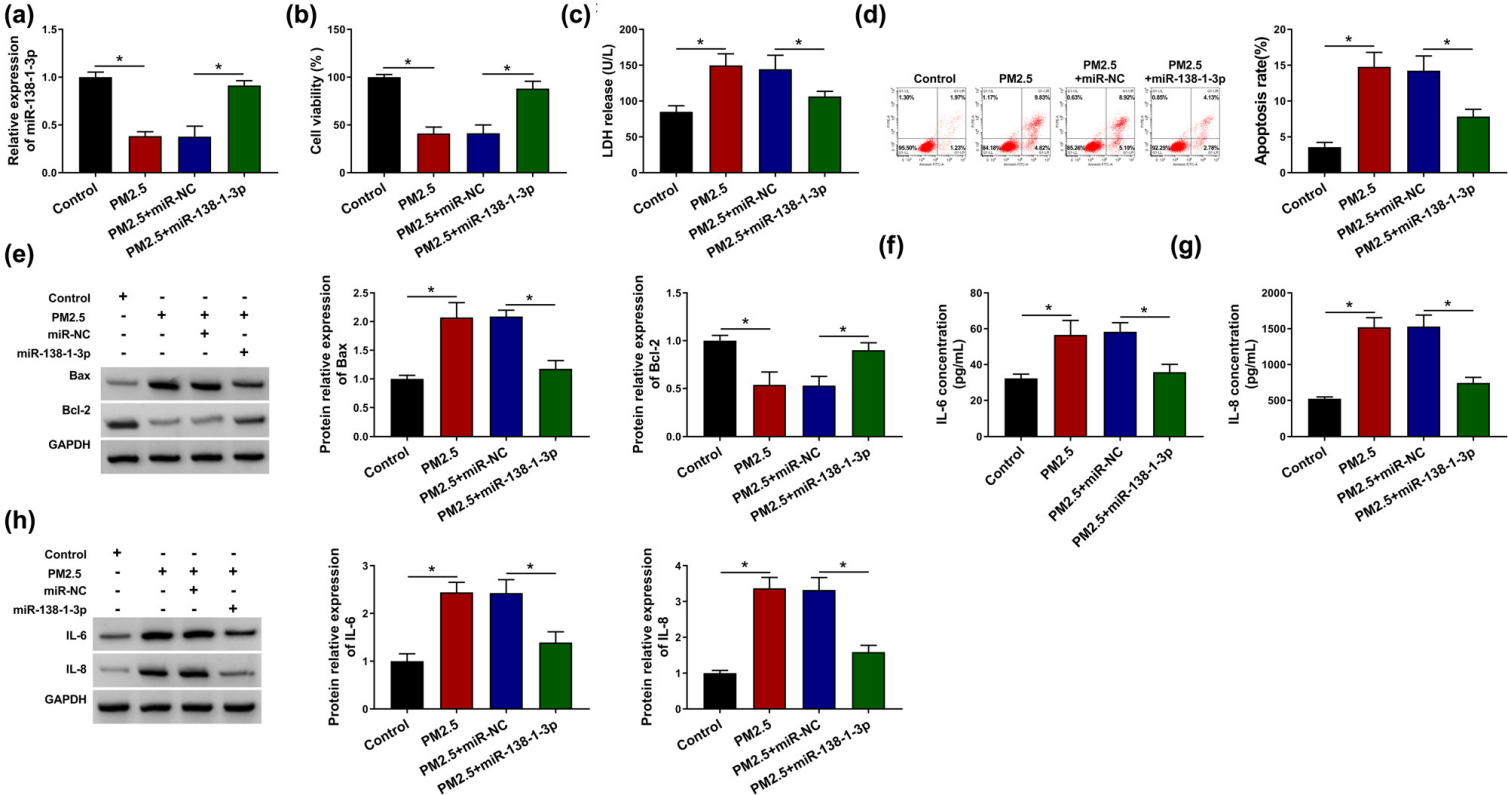
miR-138-1-3p alleviated PM2.5-induced cell death and inflammatory response in BEAS-2B cells. BEAS-2B cells were transfected with miR-NC or miR-138-1-3p, followed by treatment with 75 μg/mL PM2.5 for 48 h. (a) RT-qPCR analysis of miR-138-1-3p expression in BEAS-2B cells. (b) BEAS-2B cell viability analysis using CCK-8 assay. (c) LDH release detection in BEAS-2B cells using LDH assay. (d) Flow cytometry of BEAS-2B cell apoptosis. (e) Western blot analysis of Bax and Bcl-2 expression in BEAS-2B cells. (f–h) Levels detection of IL-6 and IL-8 expression in BEAS-2B cells using ELISA and western blot analyses. *N* = 3, *P < 0.05.

Given the decrease of miR-138-1-3p expression in PM2.5-induced BEAS-2B cells, the detailed functions of miR-138-1-3p in PM2.5-induced BEAS-2B cell injury were investigated. First, miR-NC or miR-138-1-3p was transfected into BEAS-2B cells, followed by treatment with 75 μg/mL PM2.5 for 48 h, and then we found miR-138-1-3p transfection significantly rescued PM2.5-induced miR-138-1-3p downregulation in BEAS-2B cells (Figure 4a). Next, functional experiments were conducted; results exhibited miR-138-1-3p restoration reversed PM2.5-induced cell viability suppression (Figure 4b), LDH release (Figure 4c), apoptosis promotion (Figure 4d and e), as well as the increase of IL-6 and IL-8 expression (Figure 4f–h) in BEAS-2B cells. Altogether, miR-138-1-3p reduced PM2.5-induced cell death and inflammatory response of BEAS-2B cells.

### 3.5 circ_0038467 knockdown reduced PM2.5-induced cell death and inflammatory response in BEAS-2B cells via miR-138-1-3p

**Figure 5:**
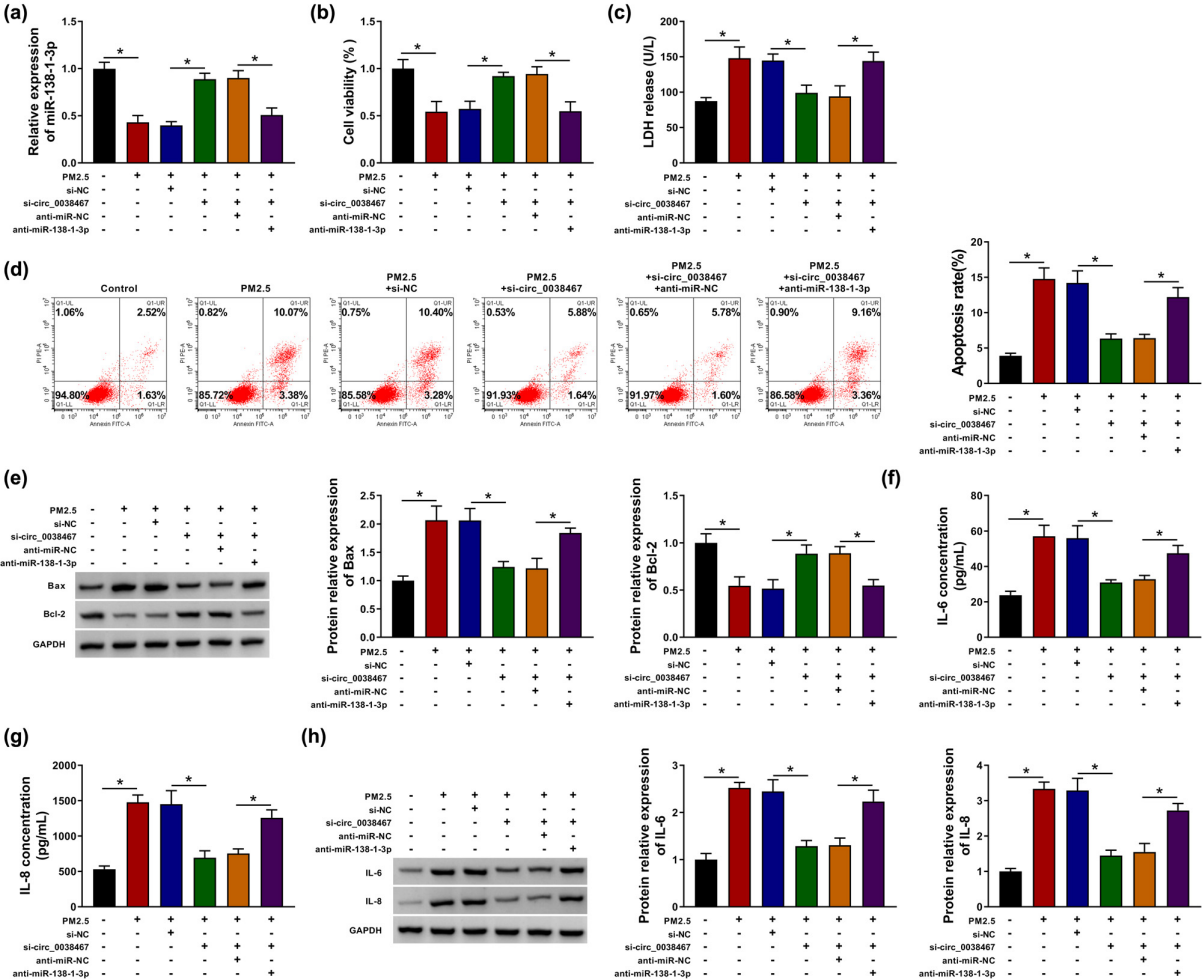
circ_0038467 knockdown reduced PM2.5-induced cell death and inflammatory response in BEAS-2B cells via miR-138-1-3p. BEAS-2B cells were stably transfected with si-NC, si-circ_0038467, si-circ_0038467 + anti-miR-NC, or si-circ_0038467 + anti-miR-138-1-3p, and then treated with 75 μg/mL PM2.5 for 48 h. (a) RT-qPCR analysis of miR-138-1-3p expression in BEAS-2B cells. (b) BEAS-2B cell viability analysis using CCK-8 assay. (c) LDH release detection in BEAS-2B cells using LDH assay. (d) Apoptosis analysis in BEAS-2B cells with flow cytometry. (e) Levels detection of Bax and Bcl-2 in BEAS-2B cells using western blot analysis. (f–h) Levels measurement of IL-6 and IL-8 expression in BEAS-2B cells using ELISA and western blot analyses. *N* = 3, *P < 0.05.

Considering that miR-138-1-3p was a target of circ_0038467, we then tested whether miR-138-1-3p was involved in the effects of circ_0038467 knockdown on PM2.5-induced cell injury. BEAS-2B cells were stably transfected with si-NC, si-circ_0038467, si-circ_0038467 + anti-miR-NC, or si-circ_0038467 + anti-miR-138-1-3p, and then treated with 75 μg/mL PM2.5 for 48 h. The results showed that transfection of anti-miR-138-1-3p significantly decreased circ_0038467 knockdown-induced upregulation of miR-138-1-3p expression in PM2.5-treated BEAS-2B cells (Figure 5a). Then rescue assay was conducted; we observed that downregulation of miR-138-1-3p partially overturned circ_0038467 knockdown-mediated cell viability enhancement (Figure 5b), LDH release (Figure 5c), and apoptosis (Figure 5d and e) inhibition, as well as IL-6 and IL-8 expression decrease (Figure 5f–h) in PM2.5-treated BEAS-2B cells. Collectively, these results suggested circ_0038467 knockdown performed protective functions in PM2.5-induced BEAS-2B cells via miR-138-1-3p.

### 3.6 PM2.5 induced the activation of nuclear factor kappa B (NF-κB) pathway through circ_0038467/miR-138-1-3p axis

**Figure 6:**
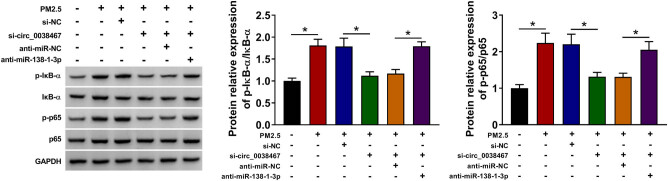
PM2.5 induced the activation of NF-κB pathway through circ_0038467/miR-138-1-3p axis. Western blot analysis of p-IκB-α and p-p65 levels in BEAS-2B cells transfected with si-NC, si-circ_0038467, si-circ_0038467 + anti-miR-NC, or si-circ_0038467 + anti-miR-138-1-3p under PM2.5 exposure. *N* = 3, *P < 0.05.

To explore whether NF-κB pathway was involved in the regulation of the circ_0038467/miR-138-1-3p axis in PM2.5-induced BEAS-2B cell dysfunction, the expression of p-IκB-α and p-p65 was measured in transfected BEAS-2B cells through western blot analysis. Results showed PM2.5 resulted in a significant increase of p-IκB-α and p-p65 levels, which were reversed by circ_0038467 knockdown; furthermore, it was also observed that silencing miR-138-1-3p significantly attenuated the inhibitory effects of circ_0038467 knockdown on p-IκB-α and p-p65 expression under PM2.5 treatment (Figure 6). Thus, we demonstrated that PM2.5 activated NF-κB pathway through circ_0038467/miR-138-1-3p axis.

## 4 Discussion

Recently, inhalant PMs-induced air pollution is becoming continually intensified with the development of the heavy industries and lax control of the pollution sources, and it has been recognized that increased PM concentration in the air may directly cause growing morbidity of respiratory system [2,5]. However, the mechanisms by which PM2.5 results in lung dysfunction are still not systemically elucidated. In a series of recent studies, the role of circRNAs in PM2.5 toxic effects has begun to be identified. For example, circ_406961 was demonstrated to suppress PM2.5-evoked cytotoxicity and inflammation in HBE cells [18]. circRNA104250 was showed to enhance PM2.5-triggered inflammatory reaction in HBE cells [19]. Thus, circRNA may play significant roles in PM2.5-induced lung dysfunction.

In this study, we found PM2.5 treatment upregulated circ_0038467 expression in BEAS-2B cells. Then we transfected si-circ_0038467 into BEAS-2B cells before PM2.5 treatment, and results of functional experiments indicated circ_0038467 knockdown reversed PM2.5-induced cell viability inhibition, LDH release, as well as apoptosis promotion in BEAS-2B cells. Chronic inflammatory reaction triggered by PM2.5 deposition impairs lung function and increases the incidence of COPD and asthma [20]. This study provided experimental evidences showing that PM2.5 exposure triggered IL-8 and IL-6 expression upregulation, which was attenuated by circ_0038467 silencing. Taken together, we demonstrated that downregulation of circ_0038467 rescued lung function by blocking PM2.5-induced HBE cell death and inflammatory response.

Previous studies have documented that circRNAs can directly target microRNAs (miRNAs) by serving as miRNA sponges and subsequently abrogate the function of the corresponding miRNAs [21,22]. Given that circ_0038467 showed obvious stability and predominantly localized in the cytoplasm, we then investigated whether circ_0038467 performed regulatory effects by sponging miRNAs. Through using bioinformatics methods, we confirmed that miR-138-1-3p was a target of circ_0038467 in BEAS-2B cells. miRNAs are one of the small noncoding RNAs of about 22 nucleotides in length, which are implicated in posttranscriptional gene repression, and multiple studies have reported that miRNA alterations are associated with the development and progression of lung disease [23,24]. Several miRNAs, such as miR-486 and miR-331, have been demonstrated to inhibit PM2.5-induced cell apoptosis, oxidative stress, and inflammation in human airway epithelial cells [25,26]. However, the roles of miR-138-1-3p in PM2.5 causing lung dysfunction are still unknown.

In this work, we found miR-138-1-3p expression was decreased by PM2.5 exposure, and restoration of miR-138-1-3p suppressed PM2.5-evoked BEAS-2B cell injury via mediating cell viability promotion, LDH release reduction, and apoptosis suppression. We validated that circ_0038467 could directly bind to miR-138-1-3p, thus, whether circ_0038467-mediated regulatory functions might operate through miR-138-1-3p was tested. We observed silencing miR-138-1-3p reversed the protective functions of si-circ_0038467 in PM2.5-treated BEAS-2B cells. Altogether, we demonstrated that circ_0038467/miR-138-1-3p axis mediated the effects of PM2.5 on HBE cells.

The NF-κB pathway plays a key role in regulating important cellular behaviors, particularly, cellular growth, apoptosis, and inflammatory responses, deregulation of which is associated with cancer, inflammatory, autoimmune diseases, and so on [27,28,29]. Interestingly, recent evidence uncovered that PM2.5 exposure could activate the NF-κB pathway to induce inflammation and uncontrolled proliferation in human HBE cells, thus inducing lung dysfunction [26,30,31]. In the present study, we also found that PM2.5 exposure could activate NF-κB pathway via elevating p-IκB-α and p-p65 levels, while this condition was impaired by circ_0038467 knockdown. Importantly, we also demonstrated that miR-138-1-3p inhibition overturned the inhibitory effects of si-circ_0038467 on p-IκB-α and p-p65 expression under PM2.5 exposure. Overall, PM2.5 mediated the activation of NF-κB pathway via circ_0038467/miR-138-1-3p axis in HBE cells.

## 5 Conclusion

In summary, this study uncovered that knockdown of circ_0038467 mitigated PM2.5-induced HBE cells death and inflammation via regulating miR-138-1-3p/NF-κB pathway, providing new insights into the molecular toxicological mechanisms underlying the PM2.5 and potential therapeutic strategies for future clinic intervention in PM2.5-induced lung disorders.
